# Cultivation and Characterization of Cornea Limbal Epithelial Stem Cells on Lens Capsule in Animal Material-Free Medium

**DOI:** 10.1371/journal.pone.0047187

**Published:** 2012-10-09

**Authors:** Réka Albert, Zoltán Veréb, Krisztián Csomós, Morten C. Moe, Erik O. Johnsen, Ole Kristoffer Olstad, Bjørn Nicolaissen, Éva Rajnavölgyi, László Fésüs, András Berta, Goran Petrovski

**Affiliations:** 1 Department of Ophthalmology, Medical and Health Science Center, University of Debrecen, Debrecen, Hungary; 2 Stem Cells and Eye Research Laboratory, Department of Biochemistry and Molecular Biology, Medical and Health Science Center, University of Debrecen, Debrecen, Hungary; 3 Department of Immunology, Medical and Health Science Center, University of Debrecen, Debrecen, Hungary; 4 Centre of Eye Research, Department of Ophthalmology, Oslo University Hospital and University of Oslo, Oslo, Norway; 5 Department of Medical Biochemistry, Oslo University Hospital and University of Oslo, Oslo, Norway; University of Milan, Italy

## Abstract

A simple, reproducible, animal-material free method for cultivating and characterizing cornea limbal epithelial stem cells (LESCs) on human lens capsule (LC) was developed for future clinical transplantation. The limbal tissue explants (2×2×0.25 mm) were harvested from 77 cadavers and expanded *ex vivo* on either cell culture plates or LC in medium containing human serum as the only growth supplement. Cell outgrowth at the edge of the explants was observed within 24 hours of cultivation and achieved viable outgrowth (>97% viability as measured by MTT assay and flow cytometry) within two weeks. The outgrowing cells were examined by genome-wide microarray including markers of stemness (p63α, ABCG2, CK19, Vimentin and Integrin α9), proliferation (Ki-67), limbal epithelial cells (CK 8/18 and 14) and differentiated cornea epithelial cells (CK 3 and 12). Immunostaining revealed the non-hematopoietic, -endothelial and -mesenchymal stem cell phenotype of the LESCs and the localization of specific markers *in situ*. Cell adhesion molecules, integrins and lectin-based surface carbohydrate profiling showed a specific pattern on these cells, while colony-formation assay confirmed their clonal potency. The LESCs expressed a specific surface marker fingerprint (CD117/c-kit, CXCR4, CD144/VE-Cadherin, CD146/MCAM, CD166/ALCAM, and surface carbohydrates: WGA, ConA, RCA, PNA and AIL) which can be used for better localization of the limbal stem cell niche. In summary, we report a novel method combining the use of a medium with human serum as the only growth supplement with LC for cultivating, characterizing and expanding cornea LESCs from cadavers or alternatively from autologous donors for possible treatment of LESC deficiency.

## Introduction

Cornea limbal epithelial stem cells (LESC) have been described to exist within special niches located at the basal cell layer of the limbal epithelium at the corneo-scleral junction [1]–[4]. The role of the LESCs is in renewal of healthy [5], [6] and regeneration of injured [7], [8] corneal epithelium. Besides infection and injury, corneal diseases can also affect the LESCs and their renewal potency resulting in serious visual problems. Any imbalance of the wound-healing process can result in an increased corneal vascularization and decreased transparency [5], [9], [10].

The recovery of the corneal epithelium arises mainly from the LESCs continually giving rise to transient amplifying cells (TACs) which migrate centripetally and superficially while becoming more and more differentiated [3], [11], [12]. Some proofs of existence of corneal epithelial stem cells centripetal to the limbus have also been reported based on their colony-forming potential [13].

Due to lack of corneal donor tissue or decreased chance for graft survival after penetrating keratoplasty, an autologous or homologous expansion of human LESCs has been proposed in cases of limbal stem cell deficiency (LSCD) [10]. Many attempts have been made for using human amniotic membrane (HAM) and other bioscaffolds as carriers for transplanting LESCs [14]–[19]. The HAM has some obvious biodegradable and immunosuppressive properties during transplantation, although its thickness and variable transparency have been used as a counter argument for its use. In addition, using feeder cells or complex media containing growth factors and animal materials has raised the safety issue of transferring prion or yet unknown diseases [20], [21]. The growth of LESCs using animal-material free medium on HAM has only recently been described for transplantation purposes [22].

The use of human lens capsule (LC) as a bioscaffold for growing limbal stem-like cells has been introduced earlier [14]. We explored use of LC for *ex vivo* cultivation of LESCs in human serum as the only growth supplement due to its high transparency and small thickness, as well as low immune- or xenogenic factors insulating ability [23]. Cornea LESCs grown under such conditions were characterized by genome-wide microarray and immunostaining for markers of stemness (tumor/transformation-related protein 63 (p63/TP63), ATP-binding cassette sub-family G member 2 (ABCG2), cytokeratin (CK/KRT) 19, Vimentin (Vim) and Integrin (Itg/ITG) α9), proliferation- (Ki-67/MKI67), limbal epithelial- (CK 8/18 and 14) and differentiated corneal epithelial- (CK 3 and 12) markers [4], [22], [24]. Phenotyping covered the exclusion of hematopoietic, endothelial and mesenchymal stem cell markers [25] as well as surface Itgs, cell-adhesion molecules (CAMs) and broad lectin-based surface carbohydrate marker profiling [4], [26]. We propose a simple, reproducible, animal-material free method for *ex vivo* expansion and characterization of cornea auto- or allografts of LESCs on LC for the treatment of LSCD.

## Results

### Cultivation and Viability of Human Cornea LESCs

**Figure 1:**
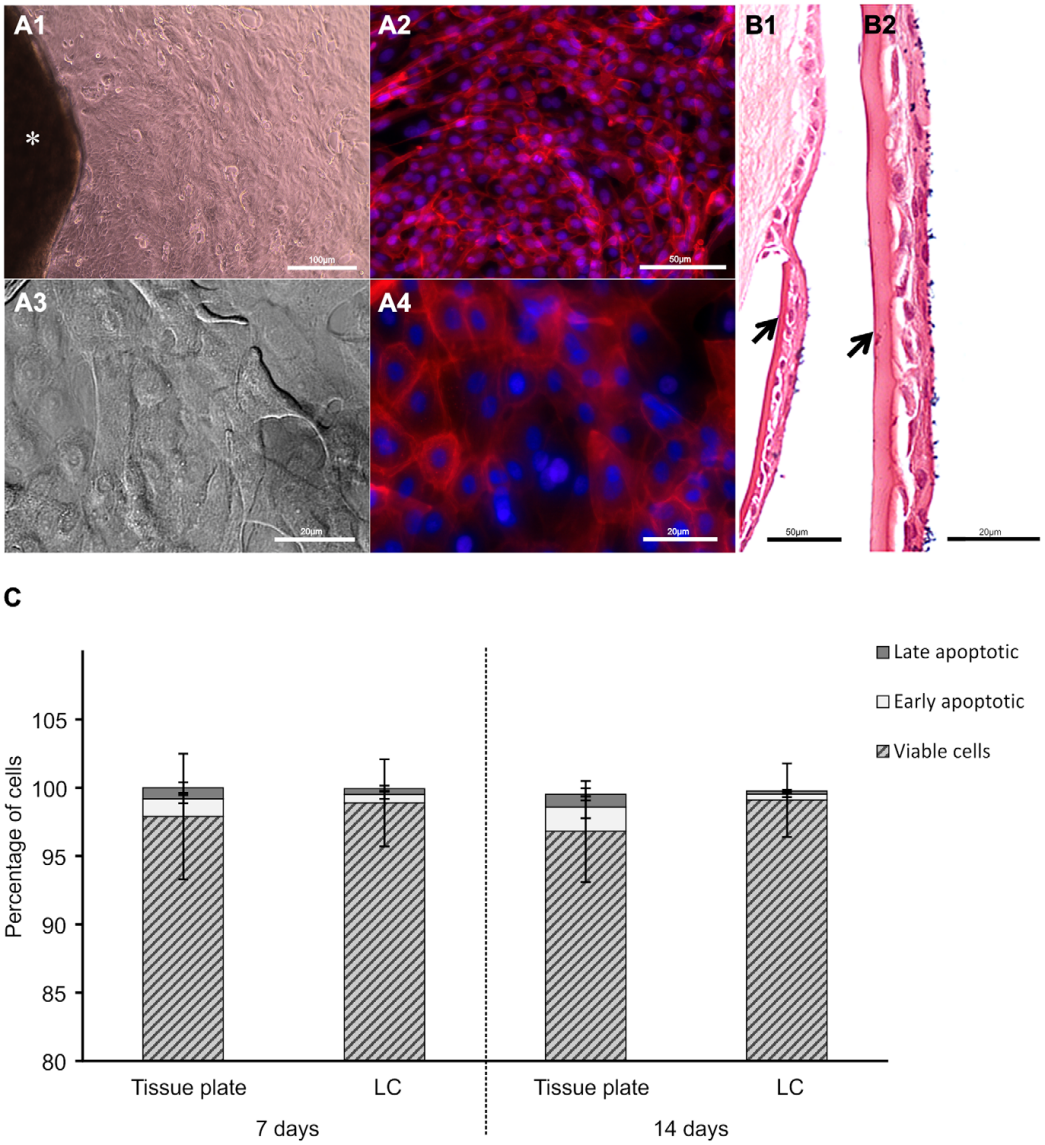
Cultivation and viability of LESCs. Limbal graft (*) cultured on cell culture plate (**A**) or human LC (**B**) showing outgrowth of cells with epithelial morphology within 24 hrs of cultivation (image shown represents a 3 day cell outgrowth, **A1** and **A3** are bright field images, **A2** and **A4** are immunofluorescent images of actin cytoskeleton (red) and nucleus (blue)). Hematoxylin & Eosin staining of LESCs grown on LC (arrows) forming stratified epithelial layer at day 7 (**B1 and B2**). Cell viability and death of the cultured LESCs (viable cells (striped bar), early apoptotic or annexin V-FITC^+^ cells (light gray bar); late apoptotic or annexin V-FITC/propidium iodide^+^ cells (dark gray bar)) (**C**). Data shown are mean ± S.D (n = 3, Scale bars: 100 µm A1, 50 µm A2, 20 µm A3–4; 50 µm B1, 20 µm B2).

Human cornea limbal tissue explants were harvested from cadavers within 12 hours from biologic death and cultured on either cell culture plates or human LC. Cell culture-plated grafts showed cell outgrowth with epithelial morphology and intact cytoskeleton within 24 hours of cultivation (**Figure 1** **A1–2**). Cell proliferation was observed over another 2 weeks till it reached confluence. Similarly, grafts grown on human LC showed cell outgrowth (**Figure 1** **A3–4**) and formed stratified epithelial layer within 7 days of cultivation (**Figure 1** **B1–2**). Under both growth conditions and use of medium containing human serum as the only growth supplement, the cell viability of the outgrowing LESCs was >97% at the two checkpoints - 7 and 14 days of cultivation, as measured by the 3-(4,5-Dimethylthiazol-2yl)-2,5-diphenyltetrazolium bromide (MTT) assay (data not shown). Accordingly, the percentage of early apoptotic (<2% annexin-Fluorescein Isothiocyanate (FITC)^+^) and late apoptotic (<1% annexin-FITC^+^/Propidium iodide^+^) cells remained low under both growth conditions (**Figure 1C**) up to day 14.

### Transcriptional Profiling in Human Cornea LESCs

**Figure 2:**
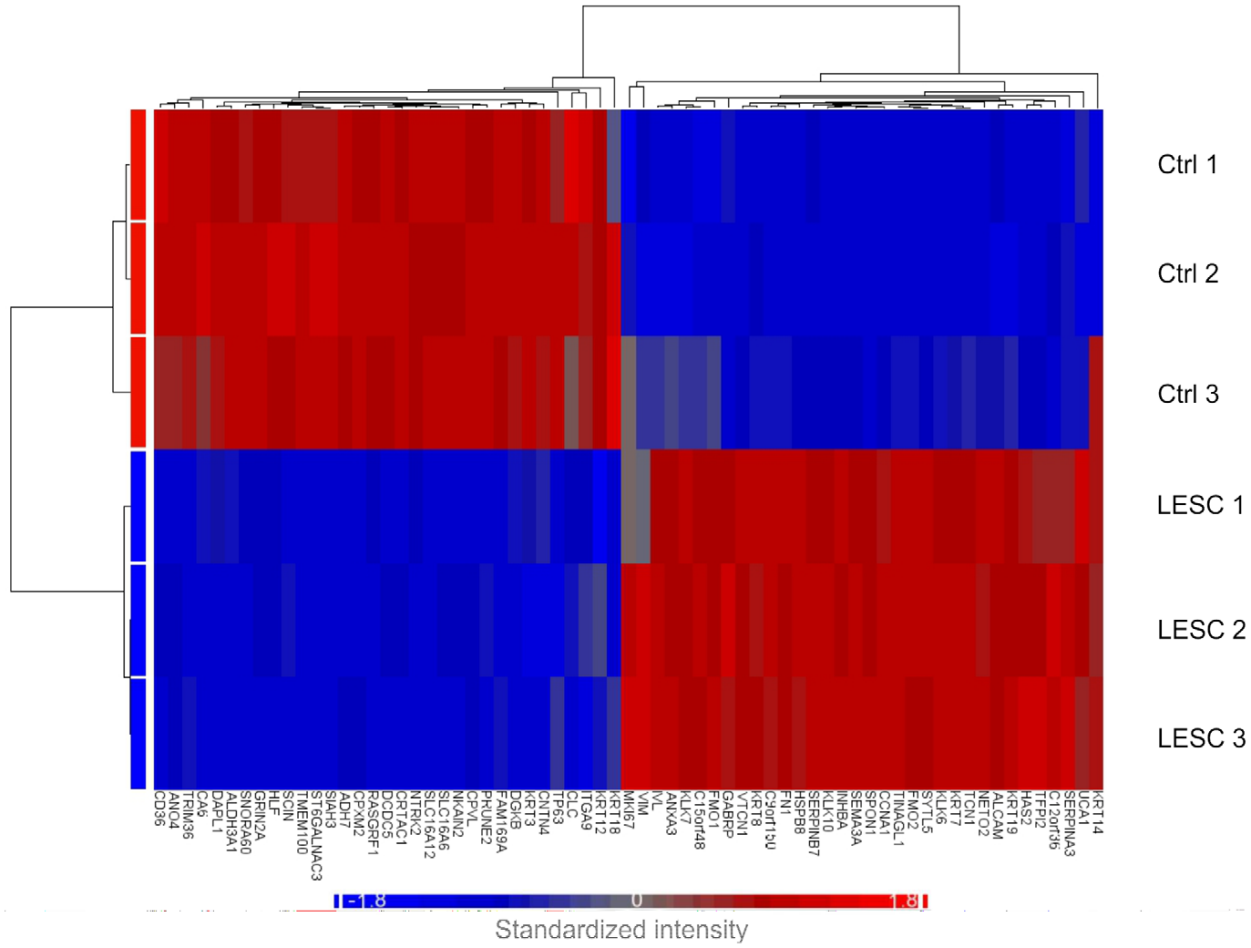
Transcriptional profiling in LESCs. Heatmap of the transcripts and functional clustering of 67 genes selected on the basis of their high or low FC or previously documented relation to LESCs (n = 3, *p*<0.01). Red and blue colors indicate high and low expression, respectively.

Transcriptional profiling of the LESCs was carried out using a microarray in three different donors. Intensity profiles of the log_2_ transformed signal values of the 28869 transcripts were obtained, out of which 955 and 875 transcripts had a more than 2 fold change (FC) increase and decrease in expression, respectively (*n* = 3, *p*<0.01), between the cultured LESCs and differentiated corneal epithelial cells. This indicates a relatively high transcriptional difference between the two cell types. **Figure 2**, **Table 1** and **Table S1** show the heatmap and the functional clustering of 67 genes selected on the basis of their high or low FC or previously documented relation to LESCs (n = 3, *p*<0.01). These genes were mostly involved in ion-, nucleotide- or protein binding, as well as receptor or enzyme activities. Among the general epithelial markers, limbal epithelium recognizing markers (KRT8/KRT18 and KRT14) could be distinguished, along the ones specific for differentiated corneal epithelium (KRT3/12). KRT8 and KRT14 showed similar or slightly higher expression levels in the limbal tissue-derived cells as compared to the differentiated control epithelium (FC: 4.0 and 1.9, respectively) indicating the commitment of LESCs towards the corneal epithelium lineage. Meanwhile, the specific differentiated corneal epithelial markers KRT3 and KRT12 decreased expression (FC: −31.0 and −5.8, respectively), probably due to an earlier differentiation state or preserved multipotency of these cells (**Table 1**). Higher expression of at least two orders of magnitude was found in the putative stem cell markers of LESCs (KRT19 (FC: 6.0) and VIM (FC: 4.4) compared to the differentiated control epithelial cells, strengthening their stem-like character. The high proliferation capacity of the cultured LESCs was also confirmed by higher expression of the proliferation-specific marker MKI67 (FC: 3.0) **(** **Table 1** **)**.

**Table 1:** Transcripts and functional clustering of selected genes in LESCs compared to differentiated corneal epithelium with high or low FC or previously documented relation to LESCs (n = 3, *p*<0.01).

Gene symbol	Gene description	Fold change	Regulation	Molecular function
KRT14	Keratin 14	2	Up	Structural constituent of cytoskeleton
SERPINA3	Serpin peptidase inhibitor, clade A (alpha-1 antiproteinase), member 3	21	Up	DNA binding
KRT19	Keratin 19	6	Up	Structural constituent of cytoskeleton
ALCAM	Activated leukocyte cell adhesion molecule	20	Up	Receptor binding
KRT7	Keratin 7	31	Up	Structural molecule activity
KLK6	Kallikrein-related peptidase 6	71	Up	Serine-type endopeptidase activity
FMO2	Flavin containing monooxygenase 2 (non-functional)	75	Up	Monooxygenase activity
SEMA3A	Sema domain, immunoglobulin domain (Ig), shortbasic domain, secreted, (semaphorin) 3A	40	Up	Receptor activity
KLK10	Kallikrein-related peptidase 10	29	Up	Serine-type endopeptidase activity
SERPINB7	Serpin peptidase inhibitor, clade B (ovalbumin),member 7	29	Up	Serine-type endopeptidase inhibitor activity
FN1	Fibronectin 1	75	Up	Extracellular matrix structural constituent
KRT8	Keratin 8	4	Up	Structural molecule activity
KLK7	Kallikrein-related peptidase 7	57	Up	Serine-type endopeptidase activity
VIM	Vimentin	4	Up	Structural constituent of cytoskeleton
MKI67	Antigen identified by monoclonal antibody Ki-67	3	Up	Nucleotide binding
KRT18	Keratin 18	1	Down	Structural molecule activity
KRT12	Keratin 12	6	Down	Structural molecule activity
ITGA9	Integrin, alpha 9	1	Down	Receptor activity
TP63	Tumor protein p63	1	Down	DNA binding
KRT3	Keratin 3	31	Down	Structural molecule activity
NTRK2	Neurotrophic tyrosine kinase, receptor, type 2	30	Down	Nucleotide binding
CRTAC1	Cartilage acidic protein 1	72	Down	Calcium ion binding
DCDC5	Doublecortin domain containing 5	43	Down	Tubulin binding
RASGRF1	Ras protein-specific guanine nucleotide-releasingfactor 1	20	Down	Guanyl-nucleotide exchange factor activity
CPXM2	Carboxypeptidase X (M14 family), member 2	25	Down	Metallocarboxypeptidase activity
ADH7	Alcohol dehydrogenase 7 (class IV), mu or sigma polypeptide	64	Down	Alcohol dehydrogenase (NAD) activity
ALDH3A1	Aldehyde dehydrogenase 3 family, member A1	30	Down	Aldehyde dehydrogenase (NAD) activity
DAPL1	Death associated protein-like 1	33	Down	Epithelial differentiation or apoptosis
CA6	Carbonic anhydrase VI	33	Down	Carbonate dehydratase activity

### Expression of Epithelial-, Stemness- and Proliferation Specific Markers in LESCs Grown on Human LC Measured by Immunofluorescence Staining

**Figure 3:**
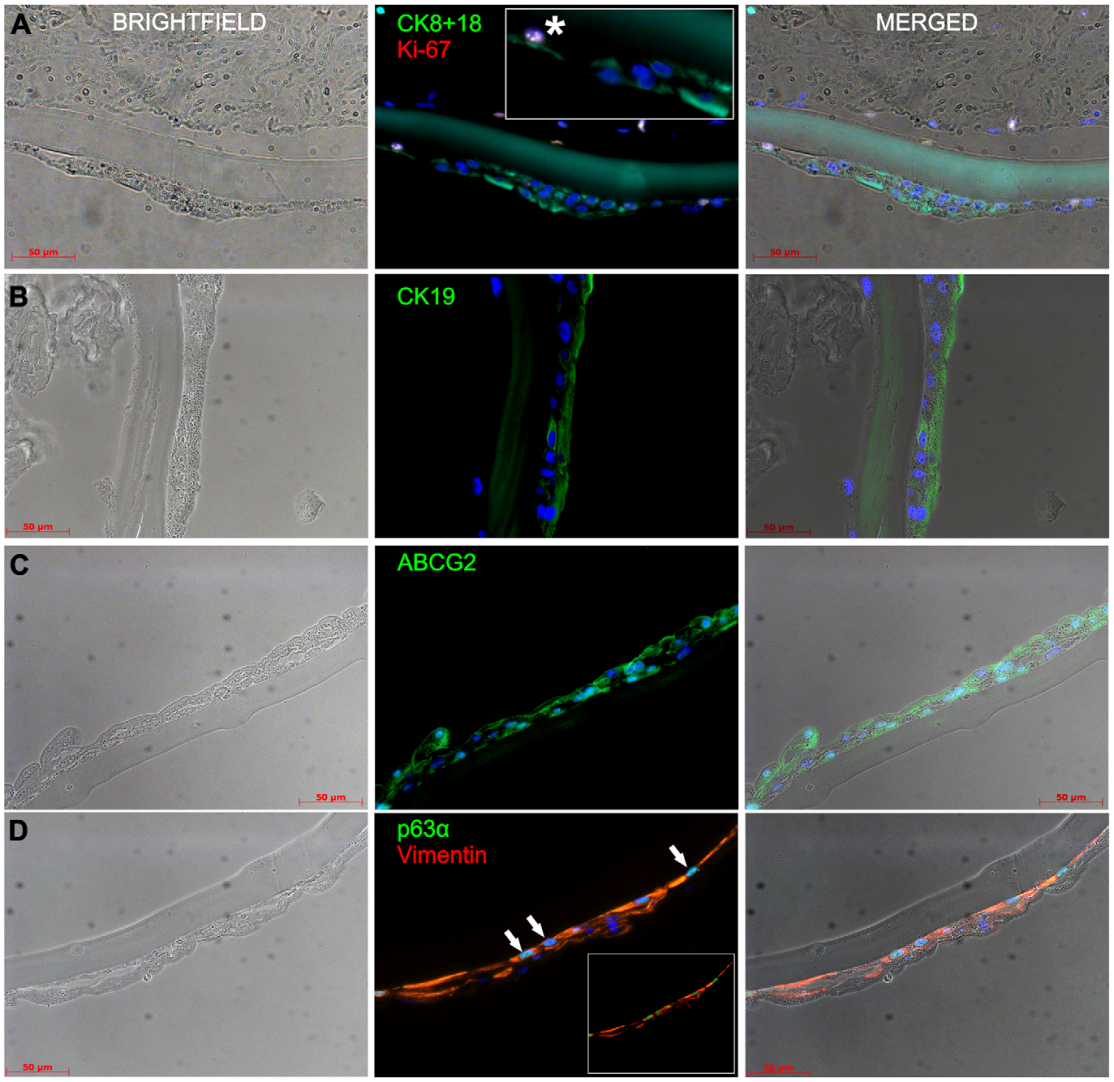
Expression of epithelial-, stemness- and proliferation specific markers in LESCs grown on human LC measured by immunoflourescence staining. Immunohistochemistry was performed to detect the (co)-expression of CK8/18/Ki-67, CK19, ABCG2, Vim/p63α in the LESCs grown on human LC (Left column: bright field-; Center: immunofluorescent; Right column: merged image; Colors on the text correspond to the color of the marker examined, while all nuclei are stained blue with DAPI; Insert: shows co-localization of CK8+18 and Ki-67 and (*) refers to the different staining pattern in the region shown; Arrows: show expression of Vimentin in the basal cells; the images are representative of at least 3 independent experiments, scale bar: 50 µm).

To validate the expression of previously identified genes at the protein level, LESCs grown on human LC were stained by fluorescent labelled specific antibodies (**Figure 3**). CK19 showed a scattered cytoplasmic staining throughout the outgrowing cell sheet, representing the corneal phenotype of the cultured cells. The expression of ABCG2, a putative marker of stemness was also observed in both the cell membrane and cytoplasm of LESCs. Strong staining and co-localization of the proliferation marker Ki-67 and CK8/18 was present in some cells (**Figure 1A** **, insert**), further confirming the proliferating and differentiating potential of these cells, respectively. The nuclear protein p63α and Vim, both markers of stemness, showed co-localization and positivity in most of the LESCs grown on human LCs.

### Phenotyping of the Cell Surface Markers on LESCs

To define the phenotype of the outgrowing cells, a flow cytometric analysis with well-known stem cell surface markers corresponding to hematopoietic, endothelial and mesenchymal lineageswas carried out on the LESCs grown on human LC (summary of the results and flow cytometry histograms are shown in **Table 2** and **Figure S1, respectively**).

**Table 2:** Expression of hematopoietic, endothelial, stemness and adhesion molecules on LESCs.

		LESC	bmMSC	
Hematopoietic	CD14	12.13±4.85	0.38±0.1	*
Monocyte markers	CD34	0±0	0±0	
	CD45	0±0	0±0	
	CD47	98.98±0.10	96.97±0.81	
	CD133	0±0	0±0	
	CD117/c-kit	17.98±4.53	0±0	*
	CXCR4	27.81±4.41	0±0	**
	HLA-DR	0±0	0±0	
Endothelial markers	CD31/PECAM	0±0	0±0	
	CD144/VE-Cadherine	81.92±3.57	41.55±9.57	*
	VEGFR2/KDR	0±0	0±0	
	CD104/Integrin β4	75.87±5.18	38.49±10.31	*
MSC	CD73	87.91±1.24	90.59±1.80	
Fibroblast markers	CD90/Thy-1	12.25±4.29	90.13±0.96	***
	CD105/Endoglin	42.09±4.91	81.90±1.96	***
	CD147/Neurothelin	97.13±0.33	75.21±7.81	
	PDGF Rβ	54.93±1.68	75.36±7.80	
Integrins and CAMs	CD29/Integrin β1	97.01±0.40	92.77±1.65	
	CD44/H-CAM	16.55±4.95	87.90±2.48	***
	CD49a/Integrin α1	71.73±6.09	71.42±7.15	
	CD49b/Integrin α2	91.16±1.27	60.55±7.19	*
	CD49f/Integrin α6	68.38±8.18	0±0	***
	CD56/NCAM	2.17±1.03	24.68±7.57	
	CD146/MCAM	82.40±3.11	87.28±2.18	
	CD166/ALCAM	98.02±0.20	86.57±6.26	

The expression of different groups of surface antigens on the LESCs was compared to those found on bmMSCs. A small population of the LESCs expressed higher CD14, CD117/c-kit, CXCR4 which are markers of special progenitor cell types. Although the LESCs expressed most of the MSC-like markers, just a minor population expressed CD90/Thy-1 and CD105 which determine the MSC phenotype. BmMSCs lack CD49f/Itg α9 which is strongly expressed on LESCs (the data represent percentage of positive cells within the total LESC culture shown as mean ± S.D., n = 7; p<0.05 *, p<0.01 **, p<0.001 ***).

No common hematopoietic cell surface markers were detected on the outgrowing cells: CD45, CD34, CD133 and human leukocyte antigens (HLA)-DR. LESCs expressed slightly, but significantly higher CD14 when compared to bone marrow derived mesenchymal stem cells (bmMSCs) (p<0.05). A small population of LESCs showed C-X-C chemokine receptor type 4 **(**CXCR4) and CD117/c-kit positivity characteristic for migrating and early progenitor or pluripotent stem cells, respectively, which is not characteristic for bmMSCs (p = 0.0059 and p = 0.0332). High CD47 expression of cultivated LESCs was similar to that of bmMSCs demonstrating the viability and immunocompetence of both cell types.

Regarding the endothelial-related markers, no CD31/Platelet endothelial cell adhesion molecule (PECAM) and vascular endothelial growth factor receptor 2 (VEGFR2)/Kinase insert domain receptor **(**KDR) could be detected, showing no endothelial-related contamination of the cell culture. When compared to bmMSCs, more cells in the LESC culture expressed CD144/vascular endothelial (VE)-Cadherin (p = 0.0321) and CD104/Itg β4 (p = 0.0458).

Significant differences were also found between LESCs and bmMSCs in the most important MSC markers: a very small population of LESCs expressed CD90/Thymocyte differentiation antigen 1 (Thy-1) and less than half of them were CD105/Endoglin positive, unlike bmMSCs (p = 0.000032 and p = 0.0006, respectively). The expression of CD73, CD147/Neurothelin and platelet-derived growth factor receptor β (PDGF-Rβ) showed no significant difference as compared to bmMSCs.

Due to their importance in the attachment of the extracellular matrix (ECM) and maintenance of growth supporting environment, next we tested the presence of CAMs and Itgs. Significant differences were found in the expression pattern of Itgs on LESCs: CD44/homing-associated cell adhesion molecule (H-CAM) was expressed at lower (p = 0.00052), while CD49b/Itg α2 (p = 0.038) and CD49f/Itg α6 (p = 0.008) at higher levels than on the surface of bmMSCs. The expression of CD29/Itg β1, CD49a/Itg α1, CD56/neural cell adhesion molecule (NCAM), CD146/melanoma cell adhesion molecule (MCAM) and CD166/activated leukocyte cell adhesion molecule (ALCAM) were similar in LESCs and bmMSCs.

To exclude a possible lens epithelium origin of some of the significantly different markers found on LESCs, surface profiling of lens epithelial cells (LECs) was carried out also. LECs similar to LESCs were CD34^−^ and CD45^−^, expressed no CD144/VE-Cadherin and showed higher CD44/H-CAM (50.43±29.28%), similar CD146/M-CAM (74.22±2.23%) or lower expression of CD166/ALCAM (74.19±46.07%) compared to cultured LESCs (n = 3). Immunostaining of human limbal sections further confirmed the presence and localization of these LESC markers *in situ* (**Table 3** and **Figure S2**).

**Table 3:** *In situ* immunohistochemical characteristics of the basal (B) and apical cells (A), and the stroma (S) in human cornea limbal sections.

Antibody specificity	B	A	S
CD34	−	−	+
CD45	−	−	+
CD144/VE-Cadherine	+	−	+
CD44/H-CAM	−	few cells	+
CD146/MCAM	few cells	−	+
CD166/ALCAM	few cells	−	+

### Profiling of the Carbohydrate Surface Markers on LESCs

**Figure 4:**
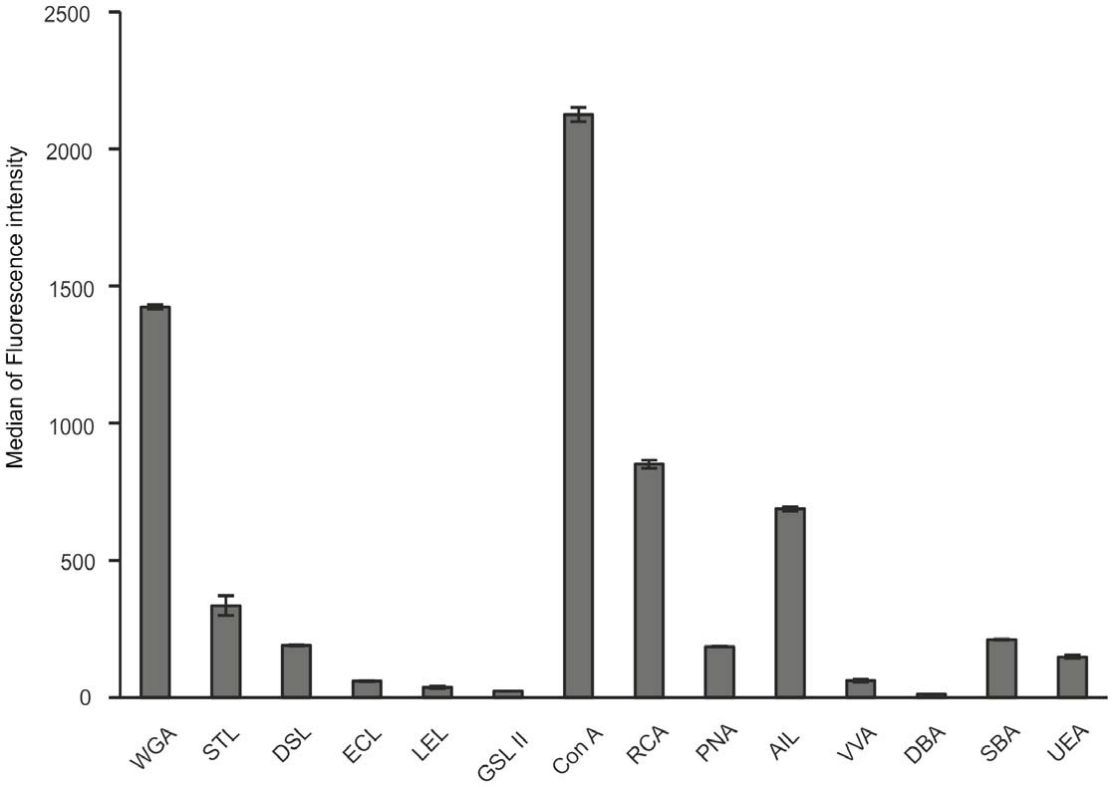
Expression of carbohydrate molecules on the surface of LESCs. Lectins-based staining of carbohydrate specific molecules on the surface of LESCs. For abbreviations used see Table 2. (Data shown are mean ± S.D. of the median of fluorescence intensity, n = 3).

The membrane of stem cells is characterized by typical carbohydrate patterns which can change during differentiation [26], [27]. Lectin-based screening of the most common terminal carbohydrates of cell surface glycolipids and glycoproteins was carried on the outgrowing LESCs **(** **Figure 4** **and** **Table 4** **)**. The surface of these cells contained high amounts of sialic acid stained by Wheat germ agglutinin (WGA) (Median = 1423.19±8.08). The majority of the cells (51.59±3.1%) showed very strong Concavalin A (ConA, Fluorescence Intensity Median (FI_med_) = 2125.02±25.99) positivity due to the presence of branched α-mannosidic structures. Ricinus communis agglutinin (RCA), Jacalin (AIL) and Peanut agglutinin (PNA), which bind to galactose and/or N-acetylgalactosamine, were all positive on LESCs, although lower fluorescence intensity could be detected by PNA (FI_med_ = 185.75±1.06) showing a small amount of T-antigen present as opposed to RCA (FI_med_ = 850.79±14.96) and AIL (FI_med_ = 687.85±7.61). Ulex europaeus agglutinin I (UEA)-lectin exhibited moderate fluorescence intensity on 61.1±1.97% of the cells, only indicating low levels of detectable fucose molecules on a subset of LESCs.

**Table 4:** Lectin-based staining of surface carbohydrate molecules on LESCs.

	Lectin	Cells (%)	Median of FI	Affinity
Sialic acid	WGA	97.37±0.33	1423.19±8.08	GlcNAcβ1–4GlcNAcβ1–4GlcNAc. Neu5Ac (sialic acid)
N-acetylglucosamine binding lectins	STL	97.68±1.64	335.38±35.63	GlcNAc oligomers
	DSL	98.94±0.23	190.04±1.23	(b-1.4) linked N-acetylglucosamine oligomers
	ECL	95.64±2.79	59.45±0.16	galactosyl (b -1.4) N-acetylglucosamine
	LEL	83.39±14.23	38.15±4.22	N-acetylglucosamine oligomers
	GSL II	82.52±2.66	24.63±0.27	alpha- or beta-linked N-acetylglucosamine
Mannose binding lectins	ConA	51.59±3.10	2125.02±26.00	high-mannose type. hybrid type and biantennary complex type N-Glycans
GalactoseN-acetylgalactosaminebinding lectins	RCA	98.28±0.35	850.79±14.96	Galβ1–4GlcNAcβ1-R
	PNA	97.92±0.51	185.75±1.06	Galβ1–3GalNAcα1-Ser/Thr (T-Antigen)
	AIL	98.99±0.01	687.85±7.61	(Sia)Galβ1–3GalNAcα1-Ser/Thr (T-Antigen)
	VVA	94.49±0.38	61.49±5.32	alpha- or beta-linked terminal N-acetylgalactosamine
	DBA	89.93±2.54	12.24±0.10	N-acetylgalactosamine
	SBA	97.11±0.60	211.11±1.32	a- or b-linked N-acetylgalactosamin
Fucose binding lectins	UEA	61.10±1.97	148.39±5.91	Fucα1–2Gal-R

The LESCs surface contained high amount of sialic acid, N-acetylglucoseamine and galactose molecules. Just around half of the cells contained mannose and two thirds contained fucose molecules, showing subpopulations within the LESC cell culture. These carbohydrate molecules determine the ECM-binding and immunological properties of the cells. ***WGA***: Wheat germ agglutinin (*Triticum vulgaris*), ***STL***: Potatoe lectin (*Solanum tuberosum*), ***DSL:*** Datura stramonium lectin (*Datura stramonium*), ***ECL:*** Erythrina cristagalli lectin (*Erythrina cristagalli*), ***LEL:*** Tomato lectin (*Lycopersicon esculentum*), ***GSL II:*** Griffonia (Bandeiraea) simplicifolia lectin II (*Griffonia simplicifolia),* ***ConA***: Concanavalin A (*Canavalia ensiformis*), ***RCA:*** Ricinus communis Agglutinin (*Ricinus communis*), ***PNA:*** Peanut agglutinin (*Arachis hypogaea*), ***AIL:*** Jacalin (*Artocarpus integrifolia*), ***VVA:*** Hairy vetch agglutinin (*Vicia villosa*), ***DBA:*** Horse gram lectin (*Dolichos biflorus*), ***SBA:*** Soy bean agglutinin (*Glycine max*), ***UEA:*** Ulex europaeus agglutinin (*Ulex europaeus*) (Data shown are mean±S.D., n = 3).

### Colony-forming Potential of LESCs

**Figure 5:**
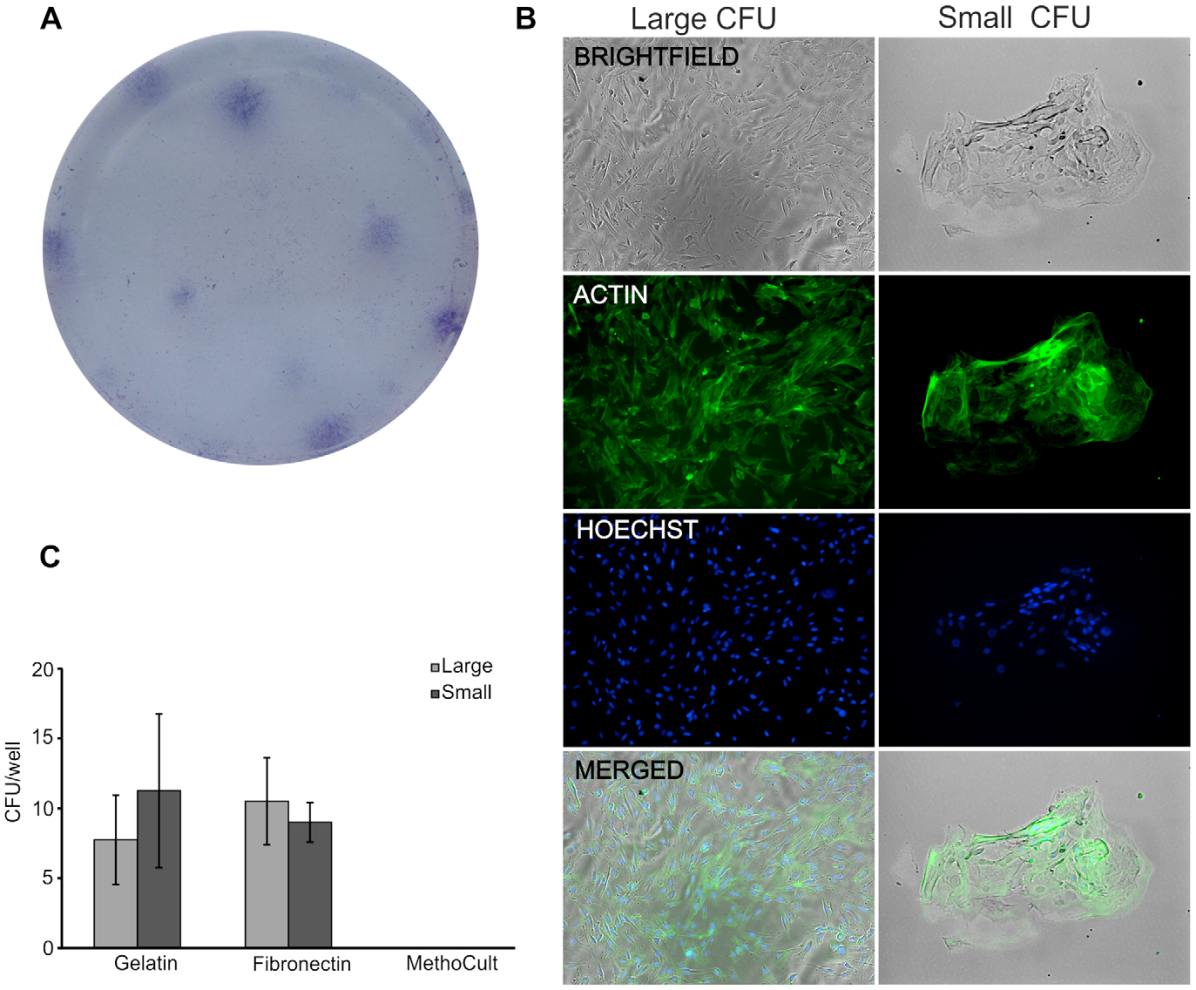
Colony-forming potential of LESCs. The LESCs were cultured at clonal density of 3000 cells/cm^2^ and early epithelial holoclone-like colony formation was recorded at day 7 of culture. LESCs formed colony forming units on Gelatin and Fibronectin coated plates as stained by crystal violet (0.5% w/v) (**A**). The colony forming unit (CFU)-forming cells were stained for actin (phalloidin-FITC, green) and Hoechst 33342 (blue, nuclear). The CFUs could be divided into two groups: large CFUs containing >50 cells, and small CFUs containing <50 LSCs. No significant difference in the CFU types on Gelatin and Fibronectin matrices was found, while MethoCult matrix-grown LESC formed no colonies at all (**B**). (Data shown are mean ± S.D., n = 3).

In order to test whether the expanded LESCs resemble the pluripotency signature reflected by the gene and protein expression levels of putative stem cell markers, their colony forming potential was tested. The LESCs were dissociated and cultivated at clonal density (3000 cells/cm^2^) on Gelatin, Fibronectin and MethoCult coated plates. All epithelial sheets tested (both cell culture plates- and human LC-grown, n = 4) were capable of forming epithelial holoclone-like colonies on Gelatin and Fibronectin as previously described [28], [29] **(** **Figure 5A** **)**. Large and small colonies were visible within 7 days of culture and exhibited active cytoskeleton and smooth-outline appearance on Gelatin and Fibronectin surfaces, but not on MethoCult coated plates **(** **Figure 5B, C** **)**.

## Discussion

An animal material-free method for *ex vivo* expansion of cornea LESCs from cadavers or autologous donors would be a safe way for tissue regeneration in cases of chemical, thermal or microbial injuries of the cornea as well as diseases such as Steven-Johnson syndrome. So far, most standard protocols for cultivating cornea LESCs included animal materials such as fetal bovine serum (FBS) and/or exogenous growth factors, hormones and cholera toxin in the growth media [30], [31]. Increasing concentrations of FBS (1–20%) have been shown to stimulate cornea limbal progenitor cells into clonal proliferation [32].

Different carriers have also been used for supporting the growth of LESCs ranging from synthetic biopolymers to natural materials such as HAM [15], anterior LC [14], fibrin matrix [17] and temperature-responsive polymers [16]. Although HAM has the advantage of containing growth, anti-angiogenic and anti-inflammatory factors that can prevent or decrease fibrosis in the healing tissue [33] or it can be sized and used as a surface for cells and biologic patch material [34], it is not transparent and thin enough.

Anterior human LC can be obtained most commonly from uneventful capsulorrhexis during cataract surgery or alternatively, from cadaver eyes at Cornea Banks. Although LC is of limited size, its transparency and thinness are superior to other biomaterials. It is the later properties upon which we decided to cultivate LESCs on human LC [14]. In addition to the previously reported use of human LC for expansion of limbal-like stem cells [14], we combined the use of such bioscaffold with the use of human serum as the only growth supplement for cultivating LESCs.

Although there are no definitive markers for identifying adult stem cells in general, or corneal stem cells in particular [35], characterization by a consensus-based panel of expression markers for LESCs [4], together with some additional, yet not described putative stem cell markers were used here for the detailed characterization of these cells.

Both cell culture plate- and LC-grown LESCs showed low death rate (<3%) over the 2 weeks cultivation period, achieving stratified epithelial-like cell growth. Presence of limbal epithelial markers CK14 and CK8/CK18 confirmed the corneo-conjunctival- and, in particular, the limbal epithelial- origin of the LESCs. CK8 being a differentiating/migrating marker also showed the transition potential of these cells towards differentiated corneal epithelium [35]–[39]. Together with the low expression of differentiated cornea epithelial markers - CK3/12 [2], [40], and the high proliferative potential of the LESCs (MKI67 expression), it can be initially concluded that the outgrowing cells are undifferentiated limbal epithelial cells.

The multipotency of the LESC was confirmed by the increased expression of putative stem cell markers: p63α [41], [42], ABCG2 [43], [44], CK19 [4], [45] and Vim [4], [45], [46]. These four markers have been described as being expressed mainly in the basal limbal epithelium [4], although Donisi et al. [47] and Sacchetti et al. [45] described lack of CK19 expression in these cells. In addition, ITG α9 has been described as being responsible for cell migration during injury [46].

Genome-wide profiling of the LESCs provided a list of high and low expressed genes that have already been demonstrated, genes that are novel or possess yet unidentified function in LESCs [48]. Serine proteinase inhibitor 3A (SERPINA3) being overexpressed in the LESCs in our gene array (FC: 21.1) has been investigated previously for its anti-angiogenic and anti-inflammatory effects during corneal injury [49]. Semaphorin 3A (SEMA3A) (FC: 40.2) has been shown to be involved in the development of mouse cornea and differentiation of cornea epithelial cells [50]. Fibronectin 1 (FN1) (FC: 74.9) is involved in cell adhesion and migration processes during wound healing [51].

Considering the fact that the slow-cycling stem cells in the limbal region represent less than 10% of the limbal basal cells [52] and the finding of stem- and proliferative/migratory cell markers on our LESCs, very likely the outgrowing cells represent a mixture of stem- and TACs rather than pure limbal stem cells.

The LESCs grown on human LC are of non-hematogenous origin. They are viable cells (99% of them are CD47^+^), express markers of early pluripotency (18% are CD117/c-kit^+^) and possess migratory capacity (28% are CXCR4^+^) very much needed during resolution of corneal injuries. In addition, the outgrowing cells carry CD104/Itg β4^+^ (76%), a marker found in basal cells of limbal and corneal epithelium [53], and also, CD144/VE-Cadherin^+^ (82%), a yet not described basal limbal cell-marker. Exclusion of the markers present on the surface of both LESCs and LECs, further strengthened CD144/VE-Caderin as a new putative LESC marker, while *in situ* immunostaining of human limbal sections confirmed its presence and localization in the basal cell layer of the limbus. In addition, yet uncharacterized putative markers of LESCs could be localized in the limbal epithelium: CD44/H-CAM in the apical layer, and CD146/MCAM and CD166/ALCAM in the basal layer.

The presence of different plasma membrane Itgs tested here: α1, α2, α6 and β1, resembles a previously reported positivity found mainly in basal limbal, but also in basal corneal cells [4], [53]. In addition, previously undefined CAMs on the surface of LESCs were detected and confirmed *in situ* as putative markers of LESCs: CD44/H-CAM in the apical layer, and CD146/MCAM and CD166/ALCAM in the basal layer of the limbus.

Since our LESCs had a generally lower expression of MSC-surface markers (CD73, CD90/Thy-1, CD105/Endoglin, PDGF-Rβ) than in bmMSCs, and a distinct or significantly different pattern of CD117/c-kit, CXCR4, Itgs α2, α6 and β4 expression (**Table 2**), very likely these cells are pluripotent and capable of migration.

Indeed, the presence of pluripotent cells in the cultured LESCs could be confirmed by the formation of small and large colonies of cells with intact cytoskeleton on Gelatin and Fibronectin surfaces, and absence of colonies on MethoCult surfaces, thus excluding the hematopoietic (myeloid or erythroid) differentiation potential of these cells.

To assess and profile the carbohydrates present in glycolipids and glycoproteins on the surface of LESCs, a comprehensive lectin-based screening for 14 carbohydrate structures (1 sialic acid, 5 N-acetylglucosamines, 1 mannose, 6 galactose and 1 fucose) was carried out. To our present knowledge, limbal epithelial cells have been shown to express unsialylated galactose residues on their cell surface recognized by PNA and lack α-2,3-bound sialic acid [54]. Our LESCs had a lower median fluorescence intensity for PNA as compared to the median values of binding detected for WGA, ConA, RCA and AIL although 98% of the cells were positive for PNA.

The distinct surface marker fingerprint of the LESCs and the 5 surface carbohydrate markers (WGA, ConA, RCA, PNA and AIL) distinguished on these cells, point out a mixed population of slowly proliferating limbal stem cells and highly proliferating, migrating and potentially differentiating TACs in the outgrowth cultures. Sorting out these two cell populations and running a differential gene expression screening (currently undertaken in our lab) would probably give better insight and understanding of the function of these cells. From transplantation point of view, having a mixture of both stem-like- and undifferentiated/TACs would be a highly desirable condition towards successful corneal transplantation.

In conclusion, we hereby show that cornea LESCs can be consistently expanded *ex vivo* on human LC using a medium containing human serum as the only growth supplement. Cells isolated and cultivated in such a way are viable; they preserve their pluripotency as confirmed by their positivity for p63α, ABCG2, CK19, Vim and Itg α9, and low expression of CK3/12. The presence of differentiation properties of our cultured cells (positivity for CK8/18 and CK14) shows the directional differentiating potential into corneal epithelium *in situ*. Additional markers of pluripotency in LESC have been described here that can be added to the future recognition of these cells and as indicative factors for positive clinical outcome after corneal transplantation.

## Materials and Methods

### Limbal Tissue Harvesting

All tissue collection complied with the Guidelines of the Helsinki Declaration and was approved by the Regional and Institutional Research Ethics Committee at the University of Debrecen, Hungary (DE OEC: 3094–2010). Limbal tissue collection was done from cadavers only and Hungary follows the EU Member States' Directive 2004/23/EC on presumed consent practice for tissue collection [55]. Limbal tissue was harvested from 77 cadavers (44 males and 33 females, age 70.5±9.3 years) within 12 hours of biologic death. In brief, after a thorough povidone iodide eye wash, the conjunctiva was incised and separated from the limbal junction consequently, a 2×2×0.25 mm rectangular shape limbal graft was dissected away and towards the cornea, respectively, at the 12 o’clock position. The depth of the graft was kept superficial or within the epithelial layer; multiple grafts were collected from a single eye. The graft dissection was performed by lamellar knife placed tangential to the surface being cut.

### Culture Medium and Cultivation Conditions

Corneal epithelial cell culture medium consisted of Dulbecco-modified Eagle’s medium (DMEM, Sigma-Aldrich, St. Louis, MO, USA) supplemented with 20% human AB serum (Human serum Type AB, PAA, Pasching, Austria), 200 mM/mL L-glutamine (Sigma-Aldrich), 10,000 U/mL penicillin- 10 mg/mL streptomycin (Sigma-Aldrich). The orientation of the graft was epithelial side up in 1,91 cm^2^ tissue culture plates. Limbal tissues were proliferated *in vitro* on human lens capsules that were obtained from uneventful capsulorrhexis during cataract surgery and pretreated with 0,025% trypsin-EDTA (PAA, Pasching, Austria) (20 minutes, 37°C). For grafting on LCs, a drop of medium was used to smooth-out the capsule first and then the limbal tissue was placed in the middle of the capsule. Following adherence to the lens capsule and/or the culture plate, the graft was cultivated in total of 1 mL medium. Feeding of the cells occurred on every alternate day. The growth of the cells was monitored under phase contrast microscope regularly. Only grafts which had cell outgrowth within 24 hours were used further to decrease the chance of fibroblast contamination.

### Assay for Cell Death Analysis

Cell death was assessed by the Annexin-V-FITC Apoptosis Detection Kit (MBL, Woburn, MA, USA) according to manufacturer’s recommendations; proportion of stained Annexin-V^+^ and Annexin-V^+^/Propidium iodide^+^ cells was determined by fluorescence activated cell sorter (FACS) analysis on FACSCalibur flow cytometer (BD Biosciences Immunocytometry Systems, San Jose, CA, USA) and data were analyzed using WinMDI freeware (Joseph Trotter, La Jolla, CA,USA).

### Microarray and Data Analysis

Microarray analyses were performed using the Affymetrix GeneChip Human Gene 1.0 ST Arrays (Affymetrix, Santa Clara, CA) which contains more than 28,000 gene transcripts. 150 ng of total RNA was subjected to Ambion WT Expression Kit (Ambion) and GeneChip WT Terminal Labeling Kit (Affymetrix, Santa Clara, CA, USA) following the manufacturers’ protocols for whole genome gene expression analysis. The arrays were washed and stained using FS-450 fluidics station (Affymetrix). Signal intensities were detected by Hewlett Packard Gene Array Scanner 3000 7G (Hewlett Packard, Palo Alto, CA, USA). The scanned images were processed using GeneChip Command Console Software (AGCC) (Affymetrix) and the CEL files were imported into Partek Genomics Suite software (Partek, Inc. MO, USA). Robust microarray analysis (RMA) was applied for normalization. Gene transcripts with maximal signal values of less than 32 across all arrays were removed to filter for low and non-expressed genes, reducing the number of gene transcripts to 23190. Differentially expressed genes between groups were identified using one-way ANOVA analysis in Partek Genomics Suite Software. Clustering analysis was made using the clustering analysis module in Partek Genomics Suite Software.

### Histological and Immunofluorescent Analysis

LESCs grown on the surface of cell culture-grade glass-cover slips or human LCs, as well as full thickness cornea limbal grafts were fixed in 4% paraformaldehyde for 20 min, room temperature. The LC-grown samples and full thickness limbal grafts were dehydrated and embedded in paraffin after which 3 µm thick longitudinal sections were obtained for staining with Hematoxylin and Eosin (H&E) according to standard laboratory protocols. Alternatively, immunofluorescent labelling with anti- p63alpha, ABCG2, CK19, CK8/18, Vim and Ki-67 antibodies was used for visualization under a ZEISS Axio Observer.Z1 (ZEISS, Oberkochen, Germany) fluorescent microscope (list of primary antibodies used is provided in **Table S2**). Similarly, immunofluorescent labelling with anti- CD34, CD45, CD144/VE-Cadherine, CD144/H-CAM, CD146/MCAM and CD166/ALCAM antibodies was used for staining the full thickness limbal grafts.

### Phenotyping of Cells and Comparison to bmMSCs

To analyse the phenotype of the isolated corneal limbal cells multicolour flow cytometric analysis was used. FITC, R-phycoerythrin (PE) and allophycocyanin (APC) conjugated antibodies were used to measue the expression of CD34, CD44, CD45, CD49f/Itg α6, CD73, CD106, CD144, CD147 (all from BD Biosciences, San Jose, CA, USA); CD49a/Itg α1 (Biolegend, San Diego, CA, USA), CD14, CD29/Itg β1, CD31, CD36, CD47, CD49b/Itg α2, CD54, CD56/NCAM, CD69, CD90/Thy-1, CD104, CD105, CD117/c-kit, CD146/MCAM, CD166/ALCAM, CXCR4, HLA-DR, PDGF-Rβ, VEGFR2 (all from R&D Systems, Minneapolis, MN, USA) and CD133 molecules (Miltenyi Biotech, Gladbach, Germany) (for further details refer to **Table S2**). Samples were measured by FACSCalibur flow cytometer (BD Biosciences Immunocytometry Systems) and data were analyzed using WinMDI freeware (Joseph Trotter, La Jolla, CA, USA). For comparison, bmMSCs were used – their availability and isolation protocol were based on another unrelated study carried by V.Z. and R.E.

### Lectin Staining of Cells

Lectin screening of isolated LESCs were performed by Lectin kits from Vector Labs (Burlingame, CA). For detecting certain carbohydrate structures, the following lectins were tested: for sialic acid (***WGA***: Wheat germ agglutinin (*Triticum vulgaris*)); for N-acetylglucosamines (***STL***: Potatoe lectin (*Solanum tuberosum*), ***DSL:*** Datura stramonium lectin (*Datura stramonium*), ***ECL:*** Erythrina cristagalli lectin (*Erythrina cristagalli*), ***LEL:*** Tomato lectin (*Lycopersicon esculentum*), ***GSL II:*** Griffonia (Bandeiraea) simplicifolia lectin II (*Griffonia simplicifolia*)); for mannose (***ConA***: Concanavalin A (*Canavalia ensiformis*)); for galactose N-acetylgalactosamines (***RCA:*** Ricinus communis Agglutinin (*Ricinus communis*), ***PNA:*** Peanut agglutinin (*Arachis hypogaea*), ***AIL:*** Jacalin (*Artocarpus integrifolia*), ***VVA:*** Hairy vetch agglutinin (*Vicia villosa*), ***DBA:*** Horse gram lectin (*Dolichos biflorus*), ***SBA:*** Soy bean agglutinin (*Glycine max*)) and for fucose (***UEA:*** Ulex europaeus agglutinin (*Ulex europaeus*)).

The lectins were diluted in Lectin dilution buffer; the rest of the staining procedure was similar to the staining described for the FACS analysis.

### Colony-forming Assay

To check the colony forming properties of LESCs, cells were seeded at a 3000 cells/cm^2^ density into 6 well plates, coated with various matrices. The wells were pre-coated for 30 minutes with either 0,1% Gelatin (Sigma-Aldrich), 10 ng/mL Fibronectin (BD Biosciences) or MethoCult (Stem Cell Technologies, Vancouver, Canada) before the cells were added. Standard growth medium for the LESCs was used and changed every other day. At day 7, the samples were fixed in 4% paraformaldehyde and stained with H&E. The colonies were stained with crystal violet (0.5% w/v) against actin with phalloidin-FITC and the nucleus with Hoechst 33342. Examination was carried out under an Olympus IX81 inverted microscope with MT20 station (Olympus, Münster, Germany), and acquired and analysed by a ScanR (Olympus) software.

### Statistical Analysis

Each experiment was performed at least three times and each sample was tested in triplicates. Data are expressed as mean ± S.D. or SEM. Statistically significant differences were determined by paired student-t tests. p<0.05 *, p<0.01 **, p<0.001 ***.

## Supporting Information

Figure S1 **Histograms of the expression of hematopoietic (A), endothelial (B), stemness (C) and adhesion (D) molecules on LESCs shown in** **Table 1** **.**
(TIF)

Figure S2 ***In situ*** **immunohistochemical staining of human cornea limbal sections for the presence and localization of LESC markers found by flow cytometry.**
(TIF)

Table S1 **Additional transcripts and functional clustering of selected genes in LESCs compared to differentiated corneal epithelium with high or low FC or previously documented relation to LESCs (n = 3, p<0.01).**
(PDF)

Table S2 **Details of the antibodies used for immunohistochemistry and/or flow cytometry.**
(PDF)
