# Supplementary figures and images for: Cultivation and Characterization of Cornea Limbal Epithelial Stem Cells on Lens Capsule in Animal Material-Free Medium
Source: PLoS One. 2012 Oct 9;7(10):e47187. doi: 10.1371/journal.pone.0047187 (PMC3467238; doi:10.1371/journal.pone.0047187)

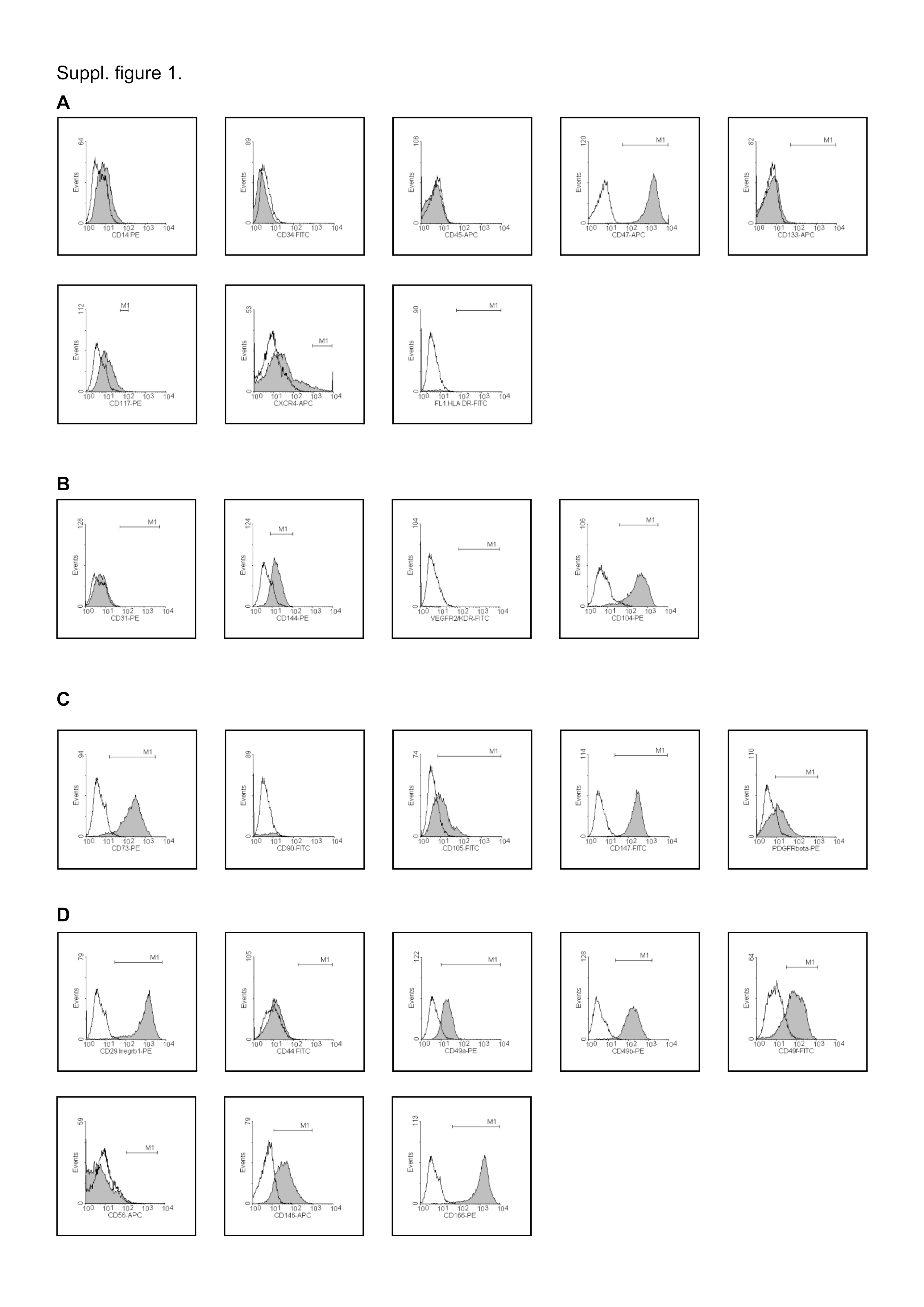

Supplement: Figure S1 — Histograms of the expression of hematopoietic (A), endothelial (B), stemness (C) and adhesion (D) molecules on LESCs shown in Table 1 . (TIF) [file pone.0047187.s001.tif]

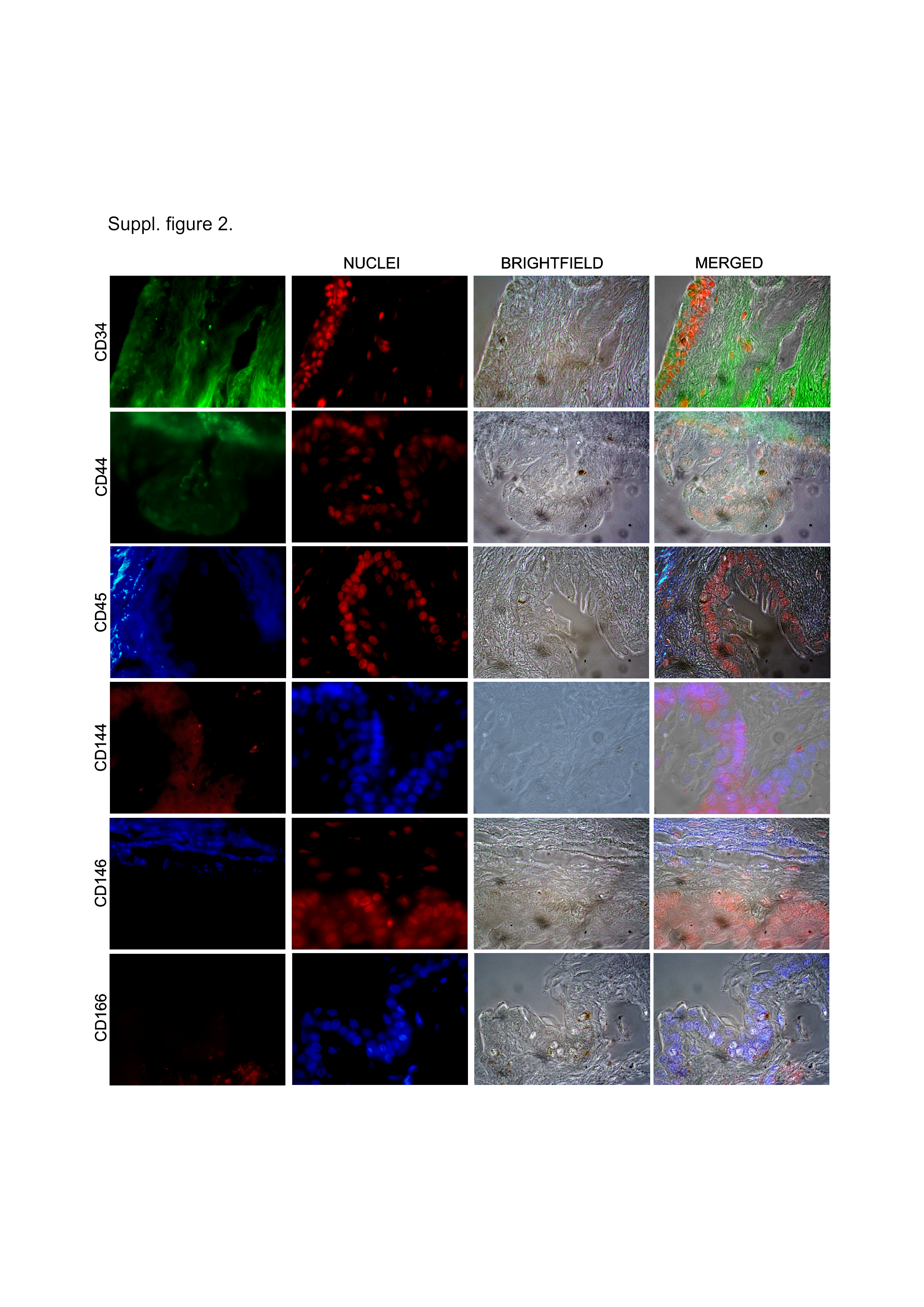

Supplement: Figure S2 — In situ immunohistochemical staining of human cornea limbal sections for the presence and localization of LESC markers found by flow cytometry. (TIF) [file pone.0047187.s002.tif]
