# Supplementary material for: Cultivation and Characterization of Cornea Limbal Epithelial Stem Cells on Lens Capsule in Animal Material-Free Medium
Source: PLoS One. 2012 Oct 9;7(10):e47187. doi: 10.1371/journal.pone.0047187 (PMC3467238; doi:10.1371/journal.pone.0047187)
Supplement: Table S1 — Additional transcripts and functional clustering of selected genes in LESCs compared to differentiated corneal epithelium with high or low FC or previously documented relation to LESCs (n = 3, p<0.01). (PDF) [file pone.0047187.s003.pdf]

Supplementary Table 1. Additional transcripts and functional clustering of selected genes in LESCs compared to differentiated corneal epithelium with high or low FC or previously documented relation to LESCs

Gene symbol	Gene description	Fold change	Regulation	Molecular function
UCA1	Urothelial cancer associated 1	21	Up	Long non-coding RNA coding sequence
C12orf36	Chromosome 12 open reading frame 36	29	Up	---
TFPI2	Tissue factor pathway inhibitor 2	24	Up	Serine-type endopeptidase inhibitor activity
HAS2	Hyaluronan synthase 2	22	Up	Hyaluronan synthase activity
NETO2	Neuropilin (NRP) and tolloid (TLL)-like 2	21	Up	Receptor activity
TCN1	Transcobalamin I (vitamin B12 binding protein, R binder family)	45	Up	Cobalamin binding
SYTL5	Synaptotagmin-like 5	26	Up	Protein binding
TINAGL1	Tubulointerstitial nephritis antigen-like 1	22	Up	Cysteine-type endopeptidase activity
CCNA1	Cyclin A1	27	Up	Protein binding
SPON1	Spondin 1, extracellular matrix protein	21	Up	Protein binding
INHBA	Inhibin, beta A	22	Up	Signal transducer activity
HSPB8	Heat shock 22kda protein 8	33	Up	Protein serine/threonine kinase activity
C9orf150	Chromosome 9 open reading frame 150	28	Up	---
VTCN1	V-set domain containing T cell activation inhibitor 1	33	Up	---
GABRP	γ-aminobutyric acid (GABA) A receptor, pi	20	Up	GABA-A receptor activity
FMO1	Flavin containing monooxygenase 1	54	Up	Monooxygenase activity
C15orf48	Chromosome 15 open reading frame 48	29	Up	---
IVL	Involucrin	20	Up	Structural molecule activity
ANXA3	Annexin A3	35	Up	Calcium ion binding
CLC	Charcot-Leyden crystal protein	20	Down	Lysophospholipase activity
CNTN4	Contactin 4	20	Down	Protein binding
DGKB	Diacylglycerol kinase, beta 90kda	43	Down	Nucleotide binding
FAM169A	Family with sequence similarity 169, member A	20	Down	---
PRUNE2	Prune homolog 2 (Drosophila)	33	Down	Oxidoreductase activity
CPVL	Carboxypeptidase, vitellogenic-like	28	Down	Serine-type carboxypeptidase activity
SLC16A6	Solute carrier family 16, member 6 (monocarboxylic acid transporter 7)	23	Down	Monocarboxylic acid transmembrane transporter activity
SLC16A12	Solute carrier family 16, member 12 (monocarboxylic acid transporter 12)	22	Down	Symporter activity
SIAH3	Seven in absentia homolog 3 (Drosophila)	24	Down	Metal ion binding
ST6GALNAC3	ST6 (α-N-acetyl-neuraminyl-2,3-β-galactosyl-1,3)-N-acetylgalactosaminide alpha-2,6-sialyltransferase 3	25	Down	Sialyltransferase activity
TMEM100	Transmembrane protein 100	21	Down	---
SCIN	Scinderin	25	Down	Phosphatidylserine binding
HLF	Hepatic leukemia factor	45	Down	DNA binding
GRIN2A	Glutamate receptor, ionotropic, N-methyl D-aspartate 2	20	Down	Receptor activity
SNORA60	Small nucleolar RNA, H/ACA box 60	19	Down	---
TRIM36	Tripartite motif-containing 36	18	Down	Protein binding
ANO4	Anoctamin 4	33	Down	Ion channel activity
CD36	CD36 molecule (thrombospondin receptor)	44	Down	Low-density lipoprotein receptor activity
