# Supplementary material for: Cultivation and Characterization of Cornea Limbal Epithelial Stem Cells on Lens Capsule in Animal Material-Free Medium
Source: PLoS One. 2012 Oct 9;7(10):e47187. doi: 10.1371/journal.pone.0047187 (PMC3467238; doi:10.1371/journal.pone.0047187)
Supplement: Table S2 — Details of the antibodies used for immunohistochemistry and/or flow cytometry. (PDF) [file pone.0047187.s004.pdf]

Supplementary Table 2

Antibody	Clone	Cat.No.	Company
ABCG2	BXP-21	b7059	Sigma
CD14	134620	FAB3832P	R&D
CD31/PECAM	9G11	FAB3567P	R&D
CD34	581	555821	BD
CD45	HI30	555485	BD
CD47	472603	FAB4670A	R&D
CD49a	TS2/7	328304	Biolegend
CD49b	HAS3	FAB1233P	R&D
CD49f	GoH3	555735	BD
CD56/NCAM	301040	FAB2408A	R&D
CD90/Thy-1		555595	BD
CD104		555720	BD
CD105/Endoglin	166707	FAB10971F	R&D
CD117/c-kit	47233	FAB332P	R&D
CD133			Miltenyi Biotech
CD144/ VE-Cadherine	55-7H1	560410	BD
CD146/MCAM	128018	FAB932A	R&D
CD147/Neurothelin	HIM6	555962	BD
CD166/ALCAM	105902	FAB6561P	R&D
CXCR4	44717	FAB173A	R&D
Cytokeratin 8/18	5D3	ab 17139	Abcam
Cytokeratin-19		rb-9021-P	NeoMarkers
HLA-DR	L203	FAB4869F	R&D
Ki-67		RM-9106-S	NeoMarkers
p63	Ab-4	Ms-1084-P	NeoMarkers
PDGF Rβ	PR7212	FAB1263P	R&D
VEGFR2/KDR	89106	FAB357P	R&D
Vimentin	SP20	RM-9120-S	NeoMarkers
